# lncRNA Metastasis-Associated Lung Adenocarcinoma Transcript 1 Promotes Proliferation and Invasion of Non-Small Cell Lung Cancer Cells via Down-Regulating *miR-202* Expression 

**DOI:** 10.22074/cellj.2020.6837

**Published:** 2019-12-15

**Authors:** Guo Tiansheng, Huang Junming, Wan Xiaoyun, Chen Peixi, Du Shaoshan, Chen Qianping

**Affiliations:** Department of Oncology, Guangzhou Panyu Hospital of Chinese Medicine, Guangzhou, PR China

**Keywords:** lncRNA-*MALAT1*, *miR-202*, Non-Small Cell Lung Cancer

## Abstract

**Objective:**

Accumulating evidences indicate that long non-coding RNAs (lncRNAs) play key roles in cancer. This study aims to clarify role of the metastasis-associated lung adenocarcinoma transcript 1 (*MALAT1*) in non-small cell lung cancer (NSCLC) and uncover the underlying mechanisms.

**Materials and Methods:**

In this experimental study, *MALAT1* and *miR-202* expression in tissues and cell lines were detected using quantitative real time polymerase chain reaction (qRT-PCR) assay. Cell transfection was conducted using Lipofectamine 3000. Cell proliferation was determined with CCK-8 assay. MMP2 and MMP9 expressions were measured with Western blot. Cell invasive ability was evaluated by Transwell assay. Starbase 2.0 tool was used to predict targets of *MALAT1*. Dual luciferase reporter assay, RNA-binding protein immunoprecipitation assay and RNA pull-down assay were conducted to confirm the potential direct interaction between *MALAT1* and *miR-202*.

**Results:**

*MALAT1* was overexpressed in NSCLC samples and cell lines. High expression of *MALAT1* was related to large tumor size (>3 cm), poor histological grade, advanced cancer and tumor metastasis in NSCLC. In vitro assays exhibited that knockdown of *MALAT1* remarkably decreased A549 cell growth and invasion capacity, while overexpression of *MALAT1* significantly enhanced NCI-H292 cell proliferation and invasion ability. Next, we verified that *MALAT1* could act as a competing endogenous RNA (ceRNA) by sponging *miR-202* in NSCLC and there is a negative correlation between *MALAT1* and *miR-202*. Besides, overexpression of miR-202 inhibited cell proliferation and invasive ability in *MALAT1*-overexpressed cells.

**Conclusion:**

This study demonstrated that lncRNA-*MALAT1* gets involved in NSCLC progression by targeting *miR- 202*, indicating that *MALAT1* may serve as a novel therapeutic target for NSCLC treatment.

## Introduction

Lung cancer is the first cause of cancer deaths worldwide, leading to about 1.6 million patients die per year (1). According to the pathological diagnosis, lung cancer is divided into small-cell lung cancer (SCLC, around 15%) and non-small cell lung cancer (NSCLC, around 85%). Although diagnostic techniques and therapy strategies (such as surgical techniques and targeted treatment) have progressed, the 5-year overall survival rate is still below 15%. Besides, this 15% of patients are accompanied with high recurrence rates (2). Thus, it is necessary to determine oncogenes involved in lung cancer development and progression and explore the underlying mechanism, facilitating development of more effective treatment methods.

Long noncoding RNAs (lncRNAs) are an emerging class of transcripts, which is longer than 200 nucleotides (nt). Although lncRNAs are coded by the genome, they are hardly translated into proteins. Previous researches revealed that lncRNAs serve as new regulators, controlling gene expressions epigenetically and post-transcriptionally. They also play crucial roles in modulating chromatin dynamics, cell growth, differentiation and development (3). Increasing evidences indicated that many lncRNAs are observed to be abnormally expressed in many types of cancer (4). For instance, Wei and Wang (5) found that lncRNA-*MEG3* was downregulated in gastric carcinoma specimens and overexpression of it could repress gastric cancer cell growth and mobility via elevating p53 expression. lncRNA *CPS1-IT1* was reported to serve as tumor suppressor in colorectal cancer and low *CPS1-IT1* expression indicated poor prognosis (6).

The metastasis-associated lung adenocarcinoma transcript 1 (*MALAT1*), also called as nuclear-enriched abundant transcript 2 *(NEAT2), HCN, LINC00047, NCRN00047* and *PRO2853*, is an extensively expressed lncRNA, with the length of around 8000 nt (7). In 2003, *MALAT1* was first found to function as a survival prognostic factor for stage I lung adenocarcinoma or squamous cell carcinoma patients (8). In recent years, accumulating evidences suggested that *MALAT1* plays a key role in tumorigenesis. In gastric cancer, *MALAT1* was reported to promote tumorigenicity and metastasis through facilitating vasculogenic mimicry and angiogenesis (9). In triple-negative breast cancer, *MALAT1* was found to promote cell proliferation and invasion via decreasing expression of* miR-129-5p* (10). Xie et al. (11) revealed that *MALAT1* suppressed apoptosis and enhanced cell invasion ability via inhibiting miR-125p in bladder cancer. In epithelial ovarian cancer, *MALAT1* was found to facilitate cell growth and induce epithelial-mesenchymal transition (EMT) through modulating PI3K/AKT signaling pathway (12). The study performed by Li et al. (13) showed that *MALAT1* is positively correlated with chemoresistance in colorectal cancer patients. Nevertheless, further investigations are still required to identify role and function of *MALAT1* in development and progression of NSCLC.

Previous studies have identified* miR-202* as a tumor suppressor. For instance, in papillary thyroid carcinoma, *miR-202* attenuates cell migration and invasion abilities via inhibiting Wnt signaling pathway (14). In human bladder cancer, *miR-202* suppresses cell growth and metastasis through targeting EGFR (15). Furthermore, *miR-202* was found to reduce expression level of TGFβ receptors and reverse TGFβ1-mediated EMT in pancreatic cancer (16). In NSCLC, *miR-202* decreased cell viability and weakens cell mobility and invasive capacity by suppressing STAT3 activity (17).

In this study, we observed that lncRNA-*MALAT1* was highly expressed in NSCLC tissues and cell lines. Correlation analysis revealed that high *MALAT1* expression was related to large tumor size (> 3 cm), moderate or poor differentiation, advanced tumor stage and metastasis. Biologically functional experiments demonstrated that *MALAT1* promoted NSCLC cell proliferation and invasion. Further molecular mechanisms revealed that *MALAT1* could sponge *miR-202* within NSCLC progression.

## Materials and Methods

### Patients and tissue samples

Forthy NSCLC tissues as well as corresponding adjacent normal tissues specimens were collected from Guangzhou Panyu Hospital of Chinese Medicine between June 2015 and July 2018. Patients involved in this study had not received any preoperative radiotherapy or chemotherapy. All specimens were identified as NSCLC tissues or normal lung tissues via histopathological observation. After resection, all tissues were dipped in liquid nitrogen promptly and then were stored at -80˚C for further studies. All enrolled patients were informed to sign the written informed consent and this study was approved by the Ethics Committees of Guangzhou Panyu Hospital of Chinese Medicine (license number of ethics statement: 2015HW126).

### Cell culture

In this experimental study, normal lung cell BEAS- 2B, NSCLC cell lines (A549, NCI-H23, NCI-H292, NCI-H1299 and NCI-H1975) and HEK293T cell were obtained from ATCC. BEAS-2B cell was cultured in BEBM medium (Lonza/Clonetics Corporation, Switzerland) containing 10% fetal bovine serum (FBS, Thermo Fisher Scientific, USA). NSCLC cell lines and HEK293T cell were cultured in RPMI-1640 medium (Thermo Fisher Scientific, USA) supplemented with 10% (v/v) FBS. All cells were maintained in a humidified atmosphere with 5% CO_2_ at 37˚C.

### RNA extraction and quantitative real time polymerase chain reaction assay

Total RNA was extracted from tissue specimens and cell lines by using TRIzol reagent (Invitrogen, USA) according to manufacturer’s protocol and treated with DNase I (Thermo Fisher Scientific, USA) to remove genomic DNA. cDNA was synthesized with the Transcriptor First Strand cDNA Synthesis Kit (Roche, Switzerland). For miRNAs, reverse transcription was conducted with TaqMan Micro-RNA Reverse Transcription Kit (Applied Biosystems, USA). Expression level of lncRNA-*MALAT1* was analyzed on a CFX96 real-time thermocycler (BioRad, USA) by using SsoAdvanced™ Universal SYBR® Green Supermix (BioRad, USA). Detection of miR-202 was performed using TaqMan microRNA Assay kit (Applied Biosystems, USA) on the CFX96 real-time thermocycler (BioRad, USA). *GAPDH* and *U6* were considered as endogenous control of lncRNA-*MALAT1* and miR-202 respectively. Relative expression levels were calculated by using 2^-ΔΔCT^ method. All primers used in this study are listed below:

MALAT1- F: 5′-AGTACAGCACAGTGCAGCTT-3′
  R: 5′-CCCACCAATCCCAACCGTAA-3′
GAPDH- F: 5′-GGAGCGAGATCCCTCCAAAAT-3′
  R: 5′-GGCTGTTGTCATACTTCTCATGG-3′
miR-202- F: 5′-CCTCCCAGGCTCACGAGGCT-3′
  R: 5′-GGTGCAGGTGCACTGGTGCA-3′
U6- F: 5′-GCTTCGGCAGCACATATACTAAAAT-3′
  R: 5′-CGCTTCACGAATTTGCGTGTCAT-3′.

The sequences of *MALAT1* were quoted from Zuo et al. (10). The sequences of *miR-202* were quoted from Hoffman et al. (18), while the sequences of *GAPDH* and *U6* were designed by ourselves using Pubmed.

### Cell transfection

siRNAs oligo targeting *MALAT1, miR-202 *mimics, scramble oligonucleotides and pcDNA3.1-*MALAT1* were supplied by GenePharma company (Shanghai, China). Transfection was conducted with Lipofectamine 3000 Reagent (Thermo Fisher Scientific, USA) in accordance with the manufacturer’s instruction. The sequence of siRNAs against *MALAT1* were as follows:

si-MALAT1: 5′-GAGCAAAGGAAGUGGCUUA-3′

si-NC: 5′-CGUACGCGGAAUA CUUCGAdTdT-3′.

### CCK-8 assay

At 24 hours post-transfection, 1×10^3^ cells/well were seeded in 96-well plates and cultured overnight. The cell viability was measured with CCK-8 (Beyotime Biotechnology, China) at different time of culture (0, 24, 48 and 72 hours) following the manufacturer’s instruction.

### Western blot assay

Total protein was extracted from cell pellet using RIPA lysis buffer (Thermo Fisher Scientific, USA) supplemented with protease inhibitors and phosphatase inhibitors (Roche) according to the manufacturer’s protocol. Concentration of total protein was determined by using BCA™ Protein Assay Kit (Thermo Fisher Scientific, USA). Then, 40 μg of protein per lane was separated by 8% sodium dodecyl sulfate-polyacrylamide gel electrophoresis (SDS-PAGE) and transferred to polyvinylidene fluoride (PVDF) membranes. 5% skim milk was used to block PVDF membranes for 1 hour at room temperature. Next, membranes were incubated with primary antibodies overnight at 4˚C, followed by incubation of secondary antibodies for 1 hour at room temperature. Next, protein bands were visualized using the enhanced chemiluminescence system (Bio-Rad Clarity Western ECL, USA). Primary antibodies, including MMP2 (1:1000), MMP9 (1:1000) and β-actin (1:1000) and HRP-conjugated secondary antibodies (1:5000) were obtained from Cell Signaling Technology (CST Inc., USA). β-actin was regarded as the internal control.

### Transwell invasion assay

24-well Transwell chambers were purchased from Corning (USA). After 24 hours transfection, 1×10^4^ suspended cells in 100 μl serum free medium were seeded in upper chambers smeared on Matrigel (BD Biosciences, USA). Bottom chambers were filled with 600 μl medium containing 10% FBS. After 48 hours culture, upper chambers were fixed with 4% formaldehyde and stained with 0.05% crystal violet. Then, a cotton swab was used to rub away cells on the above membrane. The invaded cells through membrane were counted using optical microscopy.

### Dual-luciferase reporter gene assay

Firstly, the full-length 3′-UTR of *MALAT1* with miR-202 binding sites was cloned into the downstream of firefly luciferase gene in pGL3 (Invitrogen, USA) to construct pGL3-*MALAT1* wild type (WT) and mutant (Mut). HEK293T cells were co-transfected with WT-*MALAT1*, Mut-*MALAT1* reporter gene plasmid or pRL-TK plasmids and miR-202 mimics or miR-NC with Lipofectamine 3000 (Thermo Fisher Scientific, USA). The pRL-TK Vector was intended for use as an internal control reporter vector and may be used in combination with any experimental reporter vector to co-transfect mammalian cells. The pRLTK Vector contains the herpes simplex virus thymidine kinase (HSV-TK) promoter to provide low to moderate levels of Renilla luciferase expression in co-transfected mammalian cells. 48 hours later, luciferase activity was determined with a dual-luciferase reporter assay system (Promega, USA).

### RNA-binding protein immunoprecipitation assay

A Magna RIP RNA binding protein immunoprecipitation kit was obtained from Millipore (Darmstadt, German) and the Ago2 antibody was purchased from Abcam (Cambridge, USA). RIP assay was conducted using the magna RIP RNA binding protein immunoprecipitation kit and Ago2 antibody in accordance with the instruction of manufacturer. qRT-PCR was used to determine expression level of co-precipitated RNAs.

### RNA pull-down assay

Biotin-labeled miR-NC and biotin-labeled *miR-202* were synthesized by GenePharma company (Shanghai, China). 48 hours after transfection with biotin-labeled miR-NC or biotin-labeled *miR-202*, the cells were collected to conduct an RNA pull-down experiment using PierceTM Magnetic RNA Protein Pull-down Kit (Thermo Fisher Scientific, USA) following the manufacturer’s instruction. lncRNA-*MALAT1* level was determined using qRT-PCR from the pull-down samples.

### Statistical analysis

Statistical analyses were processed with GraphPad Prism 6.0 software (GraphPad software, USA) and all data were expressed as mean ± standard deviation (SD). Student t test or one-way ANOVA was used to determine the differences between two groups or among multiple groups respectively. P<0.05 was considered as statistically significant.

## Results

### Overexpression of lncRNA-*MALAT1* is observed in NSCLC tissues and cell lines

**Fig 1:**
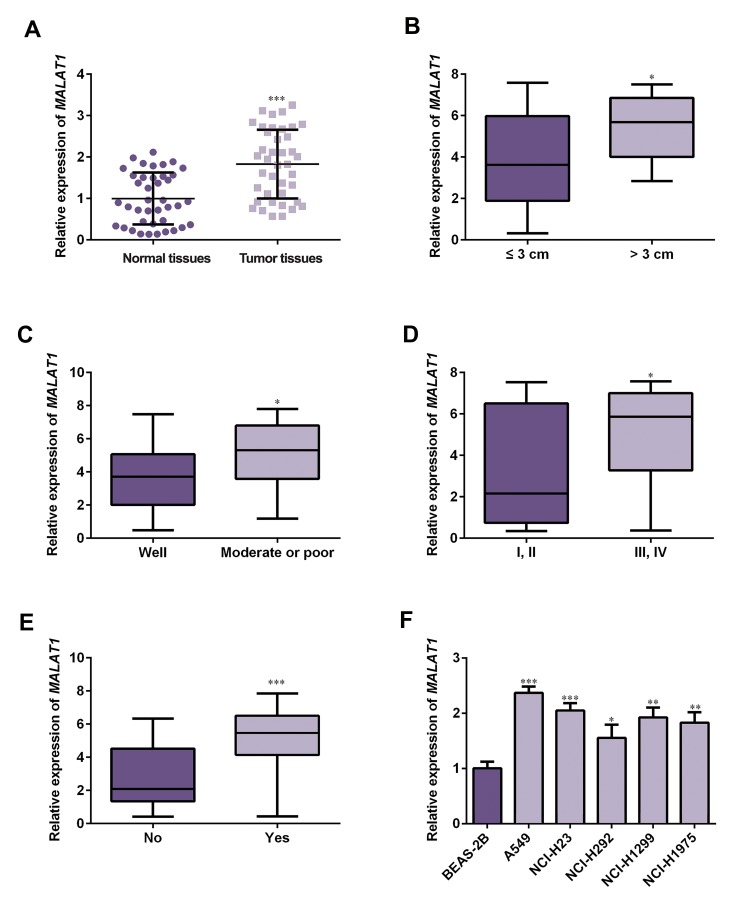
lncRNA-*MALAT1* was up-regulated in NSCLC tissues and cell lines. A. lncRNA-*MALAT1* expression in 40 paired NSCLC tissues and normal tissues was detected by qRT-PCR assay. The relationship of lncRNA-*MALAT1* expression with B. Tumor size, C. Histological grade, D. TNM stage and E. Tumor metastasis in NSCLC tissues compared to the matched paracancerous tissues (n=40). F. Expression level of lncRNA-*MALAT1* in normal lung cell BEAS-2B and NSCLC cell lines (A549, NCI-H23, NCI-H292, NCI-H1299 and NCI-H1975) was determined by qRT-PCR. *; P<0.05, **; P<0.01 and ***; P<0.001, data are expressed as mean ± SD, lncRNA; Long non-coding RNAs, NSCLC; Non-small cell lung cancer, qRT-PCR; Quantitative real time polymerase chain reaction, and TNM; Tumor nude metastasis.

To investigate the role of lncRNA-*MALAT1* in development of NSCLC carcinogenesis, we analyzed lncRNA-*MALAT1* expression in 40 paired NSCLC tissues and pericarcinomatous normal tissues with qRT-PCR. As shown in Figure 1A, the expression level of lncRNA-*MALAT1* was notably higher in NSCLC tissue samples than that in pericarcinomatous normal tissue (P<0.05). To further analyze the relationship between lncRNA-*MALAT1* expression and clinical pathological parameters, 40 NSCLC patient samples were classified into two groups in accordance with the median relative quantity of lncRNA-*MALAT1*. The lncRNA-*MALAT1* expression levels above the median expression were defined as high expression while low expression of lncRNA-*MALAT1* was termed as the expression was below the median level. Results showed that high lncRNA-*MALAT1* expression significantly associate with tumor size (>3 cm), moderate or poor differentiation carcinoma, advanced tumor stage (namely advanced TNM stage, including III and IV stages) and tumor metastases (Fig .1B-E, P<0.05). In addition, we confirmed the expression level of lncRNA-*MALAT1* in NSCLC cell lines. As shown in Figure 1F, upregulation of lncRNA-*MALAT1* was observed in NSCLC cell lines (A549, NCI-H23, NCI-H292, NCI-H1299 and NCI-H1975) compared to the normal lung cell BEAS-2B, indicating that lncRNA-*MALAT1* may play a promotor role in NSCLC. Furthermore, A549 cell expressed the highest level of lncRNA-*MALAT1* and NCI-H292 cell expressed the lowest level of lncRNA-*MALAT1*, compared to the other cell lines. Hence, A549 cell was chosen for silencing lncRNA-*MALAT1* and overexpression of lncRNA-*MALAT1* was performed on NCI-H292 cell.

### Knocking-down of lncRNA-*MALAT1* inhibits cell growth and invasion

**Fig 2:**
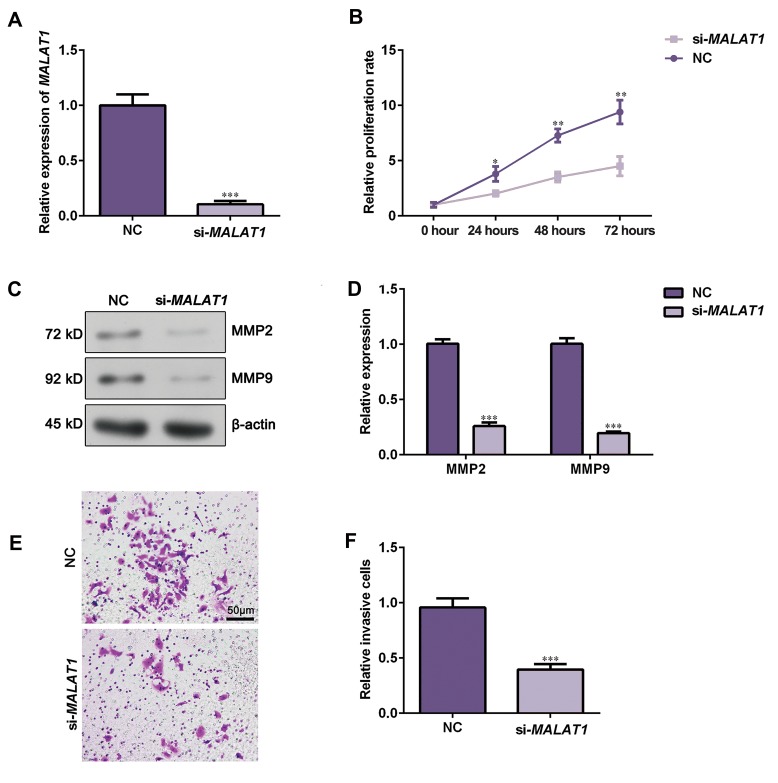
Silencing lncRNA-*MALAT1* repressed A549 cell proliferation and invasion. **A.** A549 cells were transfected with lncRNA-*MALAT1* siRNA oligo and interference efficiency was then detected by qRT-PCR. **B.** Cell viability was determined by CCK-8 assay after transfecting A549 cells with NC or si-MALAT1. **C.** Western blot assay was applied to assess MMP2 and MMP9 expression after transfection with si-MALAT1 or NC. **D.** Data represent the relative protein expression. **E.** Transwell invasion assay was applied to evaluate cell invasive potential after MALAT1 knocking-down (scale bar: 50 µm). **F.** Relative invasive cell numbers were analyzed with GraphPad Prism 5.0. Data are showed as the mean ± SD (n=3). *; P<0.05, **; P<0.01, ***; P<0.001 versus the NC group, lncRNA; Long non-coding RNAs, and qRT-PCR; Quantitative real time polymerase chain reaction.

To investigate biological function of MALAT1 in NSCLC, A549 cells were transfected with siRNAs oligo against MALAT1. As the knockdown efficiency of si-MALAT1-1 was better than that of si-MALAT1-2 (data was not shown), we silenced lncRNA-*MALAT1* expression in A549 cells by transfecting si-MALAT1-1 (Fig .2A, P<0.05). CCK-8 assay demonstrated that knockdown of lncRNA-*MALAT1* dramatically suppressed cell proliferation (Fig .2B, P<0.05). Western blot assay revealed that silencing lncRNA-*MALAT1* expression inhibited MMP2 and MMP9 expression (Fig .2C, D, P<0.05). Transwell assay presented that downregulation of lncRNA-*MALAT1* observably restrained cell invasion ability (Fig .2E, F, P<0.05). Collectively, these data demonstrated that knockdown of lncRNA-*MALAT1* inhibits A549 cell proliferation and invasion.

### Ectopic expression of lncRNA-*MALAT1* promotes cell growth and invasion

**Fig 3:**
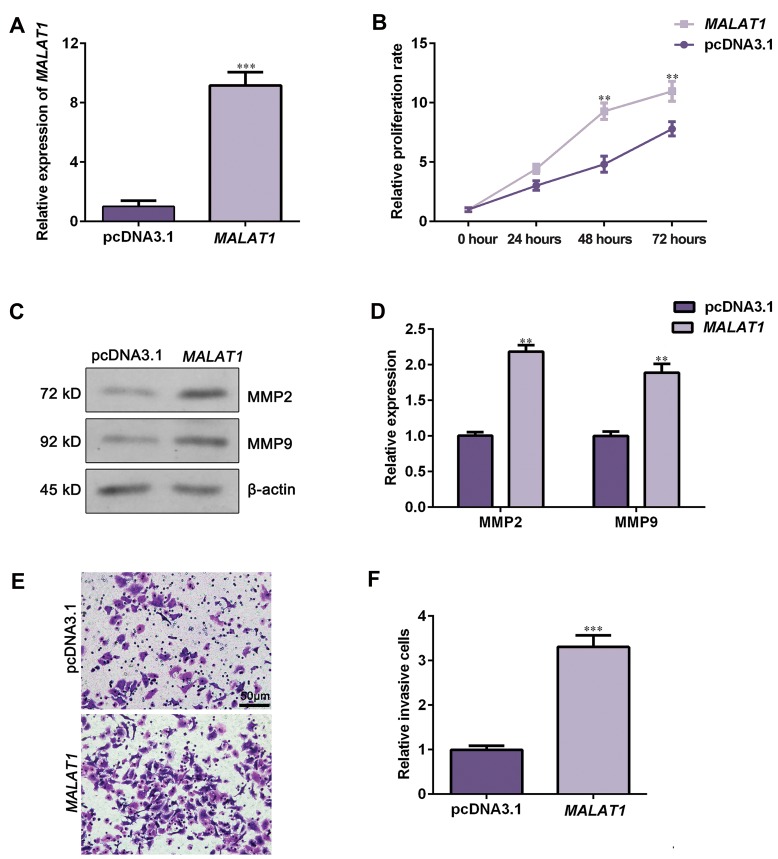
Overexpression of lncRNA-*MALAT1* promoted NCI-H292 cell proliferation and invasion. NCI-H292 cells were transfected with lncRNA-*MALAT1* plasmids or pcDNA3.1. **A.** Relative expression of MALAT1 was detected by using qRT-PCR, **B.** Cell viability was assessed by CCK-8 assay.** C.** MMP2 and MMP9 expression were evaluated using Western blot assay. **D.** Data represent relative protein expression. **E.** Cell invasion capacity was tested using Transwell invasion assay (scale bar: 50 µm). **F.** Relative invasive cell numbers were analyzed with GraphPad Prism 5.0. Data are represented as the mean ± SD (n=3). **; P<0.01 and ***; P<0.001 versus the pcDNA3.1 group, lncRNA; Long non-coding RNAs, and qRT-PCR; Quantitative real time polymerase chain reaction.

To further characterize the biological function of lncRNA-*MALAT1* in NSCLC, we established NCI-H292 cell with overexpression of lncRNA-*MALAT1* (Fig .3A, P<0.05). CCK-8 assay presented that the viability of NCI-H292 cells transfected with lncRNA-*MALAT1* plasmids was significantly increased compared to pcDNA3.1 group (Fig .3B, P<0.05). Western blot assay showed that ectopic expression of lncRNA-*MALAT1* elevated the expression of MMP2 and MMP9 (Fig.3C, D, P<0.05). Besides, Transwell assay revealed that the relative invasion capacity of NCI-H292 cells in MALAT1- overexpressed group was notably enhanced compared to pcDNA3.1 group (Fig .3E, F, P<0.05). These data further confirmed lncRNA-*MALAT1* might act as oncogene in NSCLC.

### lncRNA-MALAT1 binds to miR-202 and reduces its expression

**Fig 4:**
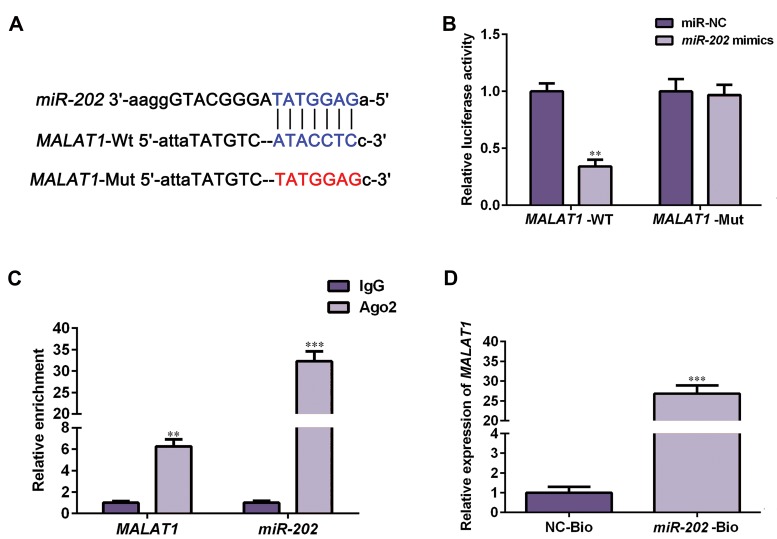
*miR-202* was a direct target of lncRNA-*MALAT1*. **A.** Starbase 2.0 (http://starbase.sysu.edu.cn) was used to identify recognition sequences between *MALAT1* and *miR-202*. **B.** NCI-H292 cells were co-transfected with *miR-202* mimics and *MALAT1*-WT or *MALAT1*-Mut, and then dual-luciferase reporter assay was employed to evaluate luciferase activity. **C.** RIP assay presented that *MALAT1* and *miR-202* expressions were enriched in Ago2 immunoprecipitates compared to IgG immunoprecipitates. **D.** RNA pull-down assay was conducted by transfecting biotin-labeled miR-NC or biotin-labeled *miR-202* into NCI-H292 cells. The endogenous expression level of lncRNA-*MALAT1* was detected by qRT-PCR. Data are expressed as the mean ± SD (n=3). **; P<0.01, ***; P<0.001, lncRNA; Long non-coding RNAs, RIP; RNA-binding protein immunoprecipitation assay, and qRT-PCR; Quantitative real time polymerase chain reaction.

As there is complementary sequence of miRNA in lncRNA, they can act as a competing endogenous RNAs, regulating miRNA expressions and biological function (19). To explore the mechanism of lncRNA-*MALAT1* in progressing NSCLC, we used bioinformatics analysis web, Starbase 2.0 (http://starbase.sysu.edu.cn), to predict targets of lncRNA-*MALAT1*. We found that lncRNAMALAT1 has a potential binding site to *miR-202* (Fig .4A). We next performed dual luciferase reporter gene assay to confirm if *miR-202* binds to the 3´-UTR of lncRNA-*MALAT1* directly. Luciferase activity was markedly attenuated in HEK293 T cells co-transfected with *MALAT1*-WT plasmids and *miR-202* mimics (P<0.05), while there was no change in the cells co-transfected with MALAT1-Mut plasmids and *miR-202* mimics (Fig .4B). This indicates that the 3´-UTR of lncRNA-*MALAT1* complementarily pairs to miR-202. Moreover, RIP assay showed that lncRNA-*MALAT1* and *miR-202* were both enriched in the Ago2 pellet compared to the IgG group (Fig .4C, P<0.05). Additionally, RNA pull-down assay presented that endogenous *MALAT1* was pulled-down specifically in the cells overexpressing miR202 compared to the NC group (Fig .4D, P<0.05). This data suggested that *miR-202* is a suppressive target of lncRNA-*MALAT1*.

### LncRNA-*MALAT1* negatively regulates *miR-202* expression in NSCLC tissues

**Fig 5:**
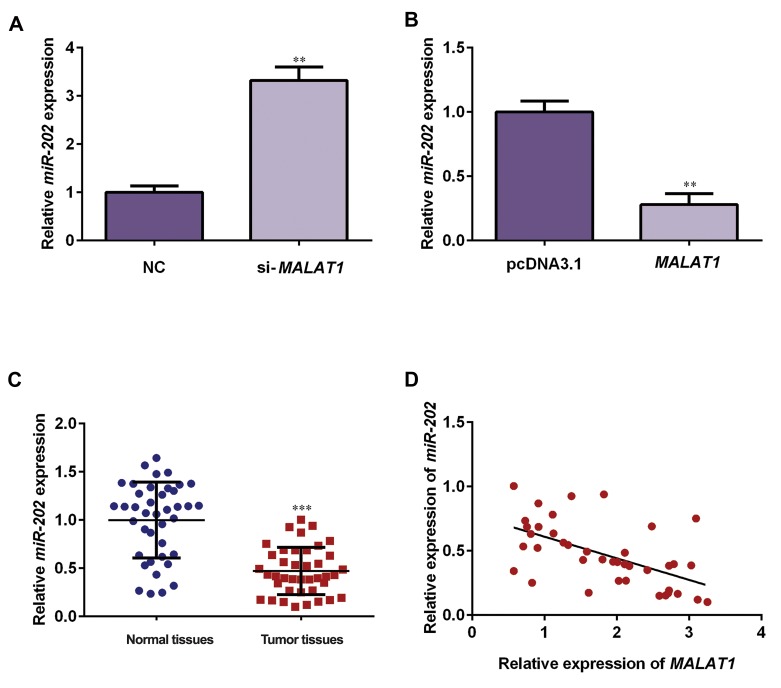
lncRNA-*MALAT1* inhibited *miR-202* expression and down-regulation of miR-202 was observed in NSCLC tissues and cell lines. **A.** A549 cells were transfected with si-*MALAT1* or si-NC, and then *miR-202* expression was determined by using qRT-PCR. **B.** NCI-H292 cells were transfected with *MALAT1*-overexpressed plasmids or pcDNA3.1, and then miR-202 expression was determined by qRT-PCR. **C.*** miR-202* expression in 40 cases of NSCLC tissues and matched paracancerous tissues was detected by qRT-PCR assay. D. Association of lncRNA-*MALAT1* with *miR-202* was assessed using Pearson’s correlation analysis (R2=0.3236, P<0.05). Relative invasive cell numbers were analyzed with GraphPad Prism 5.0. Data are showed as the mean ± SD. **; P<0.01, ***; P<0.001, lncRNA; Long non-coding RNAs, qRT-PCR; Quantitative real time polymerase chain reaction, and NSCLC; Non-small cell lung cancer.

Since lncRNA-*MALAT1* directly binds to miR-202, we next explored whether lncRNA-*MALAT1* suppresses expression of miR-202. Results of qRT-PCR assay showed that knockdown of lncRNA-*MALAT1* increased miR- 202 expression, while ectopic expression of lncRNA-*MALAT1* decreased expression level of *miR-202* (Fig .5A, B, P<0.05), suggesting that lncRNA-*MALAT1* negatively regulates miR-202. Furthermore, we observed that expression level of miR-202 was markedly downregulated in NSCLC tissues compared to the adjacent normal tissues (Fig .5C, P<0.05). We next analyzed correlation of lncRNA-*MALAT1* and *miR-202* expression levels. Findings show that* miR-202* was negatively related to the expression of lncRNA-*MALAT1* in NSCLC specimens (Fig .5D).

### lncRNA-*MALAT1* promotes NSCLC cells proliferation and invasion via decreasing *miR-202*

**Fig 6:**
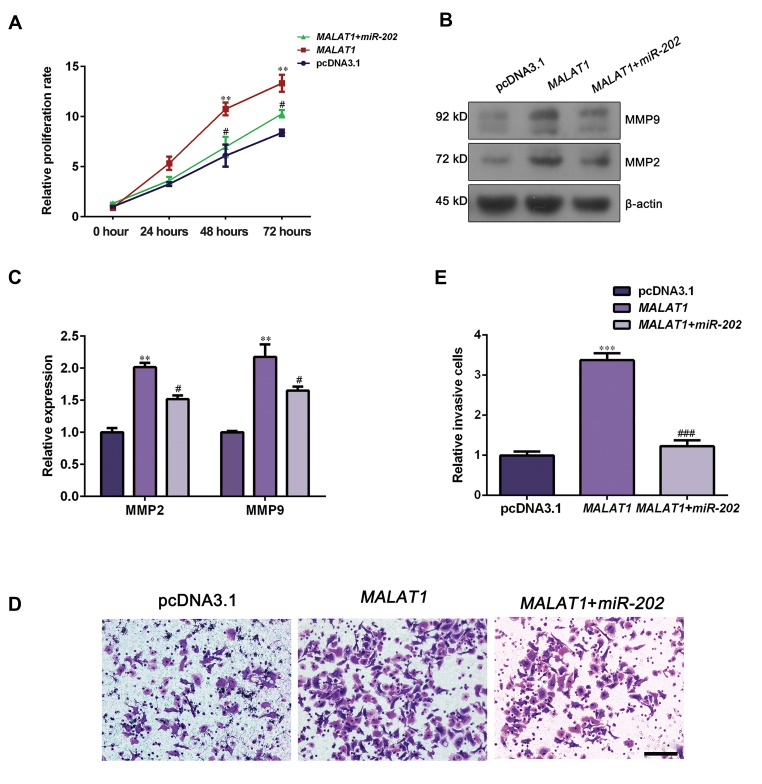
Overexpression of *miR-202* repressed cell growth and invasion in *MALAT1*-overexpressed NCI-H292 cell. NCI-H292 cells were co-transfected with *MALAT1* overexpression plasmids and *miR-202* mimics. **A.** Cell viability was detected using CCK-8 assay. **B.** MMP2 and MMP9 were determined by Western blot assay. **C.** Data represent relative protein expression. **D.** Cell invasion ability was evaluated by Transwell invasion assay (scale bar: 50 µm). **E.** Relative invasive cell numbers were analyzed with GraphPad Prism 5.0. Data are represented as the mean ± SD (n=3). **; P<0.01, ***; P<0.001 versus the pcDNA3.1 group, #; P<0.05, and ###; P<0.001 versus the *MALAT1* group.

Next, we conducted rescue experiments via overexpressing *miR-202* in *MALAT1*-overexpressed cells to investigate whether or not *miR-202* gets involved in *MALAT1*-mediated carcinogenesis. CCK- 8 assay revealed that proliferation rate of the cells cotransfected with MALAT1 and miR-202 mimics was significantly reduced compared to that of *MALAT1*- overexpressed cells (Fig .6A, P<0.05). In addition, ectopic expression of *miR-202* down-regulated expression levels of MMP2 and MMP9 in *MALAT1*- overexpressed cells, compared to *MALAT1* group (Fig .6B, C, P<0.05). In addition, Transwell invasion assay presented that *miR-202* mimics attenuated cell invasion capacity on *MALAT1*-overexpressed cells (Fig .6D, E, P<0.05). All together, these data demonstrated that lncRNA-*MALAT1* promoted NSCLC cell proliferation and invasion partially by inhibiting miR-202 expression.

## Discussion

More and more evidences have shown that the aberrant expression of lncRNAs was observed in lung cancer tissues, indicating that lncRNAs play multiple roles in carcinogenesis of lung cancer (20). For instance, Nie et al. (21) found that lncRNA urothelial carcinoma-associated 1 (*UCA1*) served as an oncogene in NSCLC. High expression of lncRNA-*UCA1* predicted short survival time and multivariate analysis indicated that *UCA1* was an independent risk parameter of prognosis. Chen et al. (22) reported that upregulation of small nucleolar RNA host gene 20 (*SNHG20*) was notably correlated with advanced tumor stage, lymph node metastases and larger tumor size, as well as poorer overall survival chance. Wang et al. (23) revealed that lncRNA-*XIST* contributed to cell proliferation and invasion by inhibition of *miR-186-5p* in NSCLC. Biological function of lncRNA-*HIT* (HOXA transcript induced by TGFβ) has been investigated in NSCLC. Results demonstrated that lncRNA-*HIT* facilitated NSCLC cell growth through interacting with E2F1 to regulate its target genes (24). Meanwhile, some lncRNAs conferring suppressive function in NSCLC have been recognized. For example, *TUG1* (taurineupregulated gene 1) functions as a tumor suppressor in NSCLC (25). Considering their roles in tumorigenesis, lncRNAs may hold promise as diagnostic or prognostic biomarkers for lung cancer.

*MALAT1* can be used to predict survival chance of stage I lung cancer or squamous cell cancer patients and it is phase and histologically specific to the metastasis of NSCLC patients (8). Accumulating studies have revealed that *MALAT1* not only plays a pivotal role in NSCLC progression, but also promotes other kinds of tumors. Zhang et al. found that serum exosome-derived lncRNAMALAT1 facilitated the tumor growth and migration, while it reduced apoptosis rate in NSCLC (26). The study performed by Li et al. (27) showed that *MALAT1* facilitated NSCLC cell growth, colony formation and apoptosis by targeting miR-124. In ovarian cancer, *MALAT1* was reported to facilitate cell proliferation and metastasis. It also prevents tumor cells from apoptosis (12). In this study, we found that *MALAT1* was overexpressed in NSCLC tissues and cell lines (A549, NCI-H23, NCI-H292, NCI-H1299 and NCI-H1975) compared to corresponding adjacent normal tissues and normal lung cell BEAS-2B, respectively. This finding was in accordance with previous studies. Further correlation analysis demonstrated that high *MALAT1* expression was positively related to large tumor size, poor histological grade, terminal stage of cancer and tumor metastasis. Knockdown of *MALAT1* inhibited A549 cell growth and invasion, as well as the expression of MMP2 and MMP9. In contrary, overexpression of *MALAT1* elevated NCI-H292 cell proliferation, invasion ability as well as the expression level of MMP2 and MMP9. These data indicated that *MALAT1* functions as oncogene in NSCLC, which is in line with previous studies.

The ceRNA theory proposes that lncRNAs sharing miRNA response elements (MREs) with mRNAs can act as miRNA decoys. It has been reported that lncRNAs can act as ceRNA by sponging miRNAs in cancer progression (28). The underlying molecular mechanisms involved in lncRNAs interacting with miRNAs are as follows: i. lncRNA indirectly inhibits negative regulation of miRNAs on target genes by competing with miRNAs to bind to the 3´-UTR of target gene mRNA, ii. Some lncRNAs form miRNA precursors by intracellular cleavage, which is then processed into specific miRNAs, regulating expression of the target genes, iii. Some lncRNAs function as endogenous miRNA sponges inhibiting miRNA expression (29). For instance, in gastric cancer, lncRNA-HOTAIR was reported to serve as a ceRNA to modulate HER2 expression via sponging miR-331-3p (30). Huang et al. (31) found that lncRNA-*CASC2 *could function as a ceRNA through sponging *miR-18a* in colorectal cancer. In the present study, targets of *MALAT1* were predicted by Starbase 2.0 (http://starbase.sysu.edu.cn). Then we used dualluciferase reporter gene assay, RIP assay as well as RNA pull-down assay to confirm that *miR-202* was a direct target of *MALAT1. MiR-202*, a new tumor suppressor, is down-regulated in gastric cancer (32). In addition, miR-202 inhibits cell growth and promotes apoptosis in osteosarcoma through decreasing expression of Gli2 (33). *miR-202* also restrains cell proliferation in human hepatocellular cancer via suppressing LRP6 expression post-transcriptionally (34). In prostate cancer, miR-202 inhibits cell proliferation and metastasis by inhibiting PIK3CA (35). Sun et al. (36) found that *miR-202* can increase therapeutic effect of cisplatin against NSCLC via inhibiting activity of the Ras/MAPK pathway. Zhao et al. (37) revealed that up-regulation of *miR-202* significantly reduces NSCLC cell viability, migration and invasion, and they suggested that STAT3 should be a direct target of *miR-202*. In this study, downregulation of miR-202 was observed in NSCLC compared to normal tissues. In addition, there was a negative correlation between *MALAT1* and *miR-202* expression in NSCLC tissues. Overexpression of *miR- 202* could reverse oncogenic effect of *MALAT1* in NSCLC, indicating that *miR-202* plays a key role in *MALAT1*-induced cell proliferation and metastasis in NSCLC cells.

The molecular mechanism whereby *MALAT1* contributes to cancer progression appears to be diverse in different cancers. In gastric cancer, *MALAT1* was found to increase cell viability via modulating SF2/ASF (38). In esophageal squamous cell carcinoma, *MALAT1* promotes cell growth and invasion via regulating ATMCHK2 signaling (39). In colorectal cancer, *MALAT1* facilitates cell growth, mobility and invasion through targeting PRKA kinase anchor protein 9 (AKAP-9) (40). In ovarian cancer, *MALAT1* contributes to cell EMT through modifying PI3K/AKT signaling pathway (12). In the present study, *MALAT1* enhances cell proliferation and metastasis by sponging miR-202 in NSCLC cell lines. To our knowledge, this is the first report revealing interaction of *MALAT1* with miR- 202 in NSCLC. However, the molecular mechanism of *miR-202* downregulation through *MALAT1* activity requires further study.

## Conclusion

This study elucidated that *MALAT1* could facilitate cell growth and invasion via sponging *miR-202* in NSCLC. Thus, our research demonstrated a new axis of *MALAT1/ miR-202*, suggesting a feasible therapeutic means for NSCLC treatment.
